# Plant-Disease-Suppressive and Growth-Promoting Activities of Endophytic and Rhizobacterial Isolates Associated with *Citrullus colocynthis*

**DOI:** 10.3390/pathogens12111275

**Published:** 2023-10-24

**Authors:** Badriya Khalfan Al-Shuaibi, Elham Ahmed Kazerooni, Shah Hussain, Rethinasamy Velazhahan, Abdullah Mohammed Al-Sadi

**Affiliations:** Department of Plant Sciences, College of Agricultural and Marine Sciences, Sultan Qaboos University, P.O. Box 34, Al-Khod 123, Oman; s121416@student.squ.edu.om (B.K.A.-S.); elham.ghasemi.k@gmail.com (E.A.K.); shahpk85@gmail.com (S.H.);

**Keywords:** antagonistic bacteria, biological control, damping-off, *Fusarium solani*, plant growth promotion, *Pythium aphanidermatum*

## Abstract

This study was conducted to investigate the antagonistic potential of endophytic and rhizospheric bacterial isolates obtained from *Citrullus colocynthis* in suppressing *Fusarium solani* and *Pythium aphanidermatum* and promoting the growth of cucumber. Molecular identification of bacterial strains associated with *C. colocynthis* confirmed that these strains belong to the *Achromobacter*, *Pantoea*, *Pseudomonas*, *Rhizobium*, *Sphingobacterium*, *Bacillus*, *Sinorhizobium*, *Staphylococcus*, *Cupriavidus*, and *Exiguobacterium* genera. A dual culture assay showed that nine of the bacterial strains exhibited antifungal activity, four of which were effective against both pathogens. Strains B27 (*Pantoea dispersa*) and B28 (*Exiguobacterium indicum*) caused the highest percentage of inhibition towards *F. solani* (48.5% and 48.1%, respectively). *P. aphanidermatum* growth was impeded by the B21 (*Bacillus cereus*, 44.7%) and B28 (*Exiguobacterium indicum*, 51.1%) strains. Scanning electron microscopy showed that the strains caused abnormality in phytopathogens’ mycelia. All of the selected bacterial strains showed good IAA production (>500 ppm). A paper towel experiment demonstrated that these strains improved the seed germination, root/shoot growth, and vigor index of cucumber seedlings. Our findings suggest that the bacterial strains from *C. colocynthis* are suppressive to *F. solani* and *P. aphanidermatum* and can promote cucumber growth. This appears to be the first study to report the efficacy of these bacterial strains from *C. colocynthis* against *F. solani* and *P. aphanidermatum*.

## 1. Introduction

Cucumber is an important crop in Oman [1]. Soilborne plant diseases represent a challenge to the cultivation and production of vegetable crops in Oman. In cucumber, damping-off is a serious problem in the USA, Canada, China, the Middle East, and other parts of the world [2,3,4,5,6,7,8]. In Oman, damping-off and decline diseases have been reported to occur in 77% of greenhouses and farms in the Al-Batinah regions and cause up to 100% losses in cucumbers and melons [9,10,11]. Soilborne diseases are also important in other vegetable crops including tomatoes, radish, and beans [12,13,14].

Soilborne diseases of cucumber are caused by a number of fungal and oomycete pathogens. *Pythium* species are among the most common soilborne pathogens affecting cucumber, with *P. aphanidermatum* being the most widespread soilborne pathogen of cucumber in Oman [11,15,16]. *Fusarium solani* and *Rhizoctonia solani* are also important soilborne pathogens of cucumber [11,17].

Management strategies of damping-off can be divided into four major categories, namely, development of resistant cultivars, chemical treatments, cultural practices, and biological control. Most soilborne pathogens, especially *Pythium*, are non-host-specific and resistant cultivars are not known yet. Chemical control is used extensively for the management of *Pythium* and other soilborne pathogens. However, systemic fungicides carry greater environmental risk [18,19] and are subject to the development of resistance [15].

Biological control (or biocontrol) indicates the use of microbial antagonists to suppress plant diseases. The biocontrol of *P. aphanidermatum* has been achieved using *Pseudomonas* sp., *Trichoderma* spp. [20,21,22], *Bacillus cereus* [23], and *B. subtilis* [24,25] isolated from various sources. Biocontrol agents are divided into two groups: endophytes, that live inside the plant; and rhizospheric microorganisms, that live attached to or beside plant roots [26,27].

A number of studies have been conducted in Oman on the biocontrol of soilborne pathogens. *Pseudomonas aeruginosa* showed antagonistic activities against *Pythium* and *Fusarium* [22,28]. The tomato rhizosphere bacteria, *Bacillus cereus* and *Exiguobacterium indicum*, and the endophytic fungus *Aspergillus terreus* isolated from the native plants *Rhazya stricta* and *Tephrosia apollinea* [29], were found to be effective against *P. aphanidermatum*. A novel fungus, *Cladosporium omanense*, and an endophytic bacterium, *Enterobacter cloaca*, showed antagonistic activity against cucumber damping-off [30,31]. Several other biocontrol agents were reported in Oman during the last 3 years, isolated from desert plants, medicinal plants, cultivated crops, and marine environments [32,33,34,35,36].

*Citrullus colocynthis* is a wild plant species belonging to the *Citrullus* genus [37]. It is a medicinal plant with several benefits [38,39]. Wild plants are known to have more resistance to diseases and tolerance to environmental stresses including drought, heat, and salinity compared to cultivated crops [40,41]. Although several studies addressed the medicinal values of *C. colocynthis*, no reports exist on the antagonistic activities of bacterial strains from this crop against *P. aphanidermatum* or *F. solani*. This study was, therefore, designed to investigate the antagonistic potential of endophytic and rhizospheric bacterial strains obtained from *Citrullus colocynthis* in suppressing *Pythium* and *Fusarium*, and in promoting cucumber growth. Knowledge in this field will help in proposing integrated management strategies for Pythium damping-off diseases.

## 2. Materials and Methods

### 2.1. Sampling

Healthy plant samples of *Citrullus colocynthis*, at the mature fruit stage, were randomly collected from Wadi Al Alaq (Al-Dakhiliya region) and Wadi Bani Khalid (Al-Sharqiya region), Oman. The two samples were collected with sterile tools and transferred to the laboratory in sterile plastic bags. The rhizospheric soil was collected from the roots by brushing the soil into sterile Petri plates. The roots, stems, leaves, and fruits were separated and the collected samples were stored in sterile containers at 4 °C. Bacterial isolation was performed within 48 h of sampling.

### 2.2. Bacteria Isolation from Citrullus colocynthis Rhizosphere and Endosphere

The isolation of cultivable bacteria was carried out as described by Ferjani et al. [42]. One gram of rhizospheric soil from each sample was added to 9 mL of sterile physiological saline solution (9 g/L NaCl), then the tube for each sample was agitated for 15 min at 200 rpm. After that, suspensions were diluted using physiological water in 10-fold series and 0.1 mL of each dilution was spread on nutrient agar (NA) culture medium (Oxoid Ltd., Basingstoke, UK).

For isolation of endophytic bacteria, the surfaces of collected samples (root, stem, leaf, and fruit) were sterilized with sodium hypochlorite (2% NaClO, 1 min), before being rinsed three times in sterile distilled water (SDW). In the next step, samples were dried out on sterile filter paper. The dried samples were either cut into small pieces with a sterile razor blade and cultured on NA medium, or they were crushed using a stomacher (Atkinson, NH, USA). The samples (10 g/sample) were placed in a sterile stomacher bag containing sterile physiological saline solution (190 mL) and homogenized for two minutes. Further, the suspensions were serially diluted and plated onto NA medium [43].

All plates were kept in an incubator for 3–7 days at 28 ± 2 °C. Different colonies were selected and purification was carried out on NA medium via sub-culturing. The bacterial strains were sorted based on phenotypic characteristics and Gram staining. Moreover, pure strains were maintained in nutrient broth (NB) (Sigma Aldrich, St. Louis, MO, USA) containing 25% glycerol at −80 °C for further study.

### 2.3. DNA Extraction, PCR Amplification, and Sequence Analysis of the Bacterial Strains

Genomic DNA of the bacterial strains was extracted from these strains using the foodproof^®^ StarPrep Two Kit (Windsor, CA, USA). The qualitative and quantitative analysis of the extracted DNA was conducted using a NanoDrop^TM^ 2000 spectrophotometer (Thermo Fisher Scientific, MA, USA). A polymerase chain reaction (PCR) was used on the extracted DNA of each bacterial strain to amplify the 16s rDNA coding region. Two universal bacterial primers, 27F (5′-AGAGTTTGATCMTGGCTCAG-3′) and 1492R (5′-TACGGYTACCTTGTTACGACTT-3′), were used for the amplification, following the described thermocycler conditions given by dos Santos, et al. [44]. The PCR reactions consisted of genomic DNA, primers, and PuRe Taq Ready-To-Go™ PCR beads (Cytiva, Marlborough, MA, USA). The PCR products were purified and sequenced by Macrogen Inc. (Seoul, Republic of Korea). The obtained sequences from this study were compared with reference sequences of closely related species in the National Center for Biotechnology Information (NCBI) database. GenBank accession numbers were allocated for the 16S ribosomal DNA sequences of the strains.

### 2.4. Screening for Antagonistic Activity

#### 2.4.1. In Vitro Antifungal Assays

All rhizospheric and endophytic bacterial strains were inspected for their antifungal activity against the cucumber fungal pathogens *Fusarium solani* and *Pythium aphanidermatum* through a dual culture assay, as described by Anith, et al. [45]. The used fungal pathogens, *F. solani* and *P. aphanidermatum*, were part of a collection of plant pathogens maintained in the plant pathology laboratory at the Department of Plant Sciences (Sultan Qaboos University, Muscat, Oman). They were cultured on potato dextrose agar (PDA) culture medium (Sigma Aldrich, MO, USA) and then a mycelial disc (5 mm diameter) was excised from the growing edge of a fungal pathogen colony (5 days old) using a sterile cork borer. The mycelial disc was placed in the middle of PDA medium inoculated with a 2-day-old bacterial strain. PDA plates inoculated with the fungal pathogen alone were considered as the control. After that, all cultures were maintained at 28 ± 2 °C for 3–7 days and the fungal growth diameter was measured from the edge of the fungal disc up to the active growing edges of the fungus compared with the control. The inhibitory impact of the bacterial strains was estimated as the inhibition percentage [45].

#### 2.4.2. Scanning Electron Microscope (SEM)

PDA plates from the dual culture assays which showed antifungal activity of the bacterial strains were chosen for electron microscopy study. The hyphal morphology of fungal pathogens (*F. solani* and *P. aphanidermatum*) on the edge of bacterial colonies was observed under SEM (JEOL JSM-5600, Tokyo, Japan). The hyphal samples of fungal pathogens were cut out and fixed in glutaraldehyde (2.5% C_5_H_8_O_2_, 4 °C, 2 h) and then washed in phosphate buffer saline (4 times). Afterwards, the samples were desiccated in a graded ethanol series (25%, 75%, 95%, and 100%) for 10 min each. Finally, they were air dried, coated with gold, and scanned using an SEM to record abnormalities in the fungal hyphae [46].

### 2.5. Determination of Growth-Promoting Potential

#### 2.5.1. Indole Acetic Acid (IAA) Quantification

The antagonistic bacterial strains were evaluated for their in vitro putative growth-promoting attribute, IAA production. The method, as adopted earlier [47], was applied to determine the IAA quantity. Strains were cultured for 72 h on NB medium supplied with 1 mM of L-tryptophan at 28 ± 2 °C. Then, the bacterial cultures were centrifuged and the supernatant was analyzed directly using high-performance liquid chromatography (HPLC) (SIL-30A, Shimadzu, Tokyo, Japan). The chromatograms from the HPLC were produced by injecting 20 µL of each filtered sample into the C_18_ HPLC column (5 µm particle size, 250 mm column length, 4.6 mm column internal diameter; Hewlett Packard Enterprise, San Jose, CA, USA). Methanol and water were used as the mobile phase at the specific ratio (80:20 *v*/*v*]), and the pH was adjusted to 3.8 using sulfuric acid. The mobile phase flow rate was 1 mL/min and the spectra scanning of the compound was performed using a photodiode array detector (Prominence SPD-M20A, Shimadzu, Tokyo, Japan) at 278 nm.

#### 2.5.2. Seed Germination Test via Paper Towel Method

**Figure 1:**
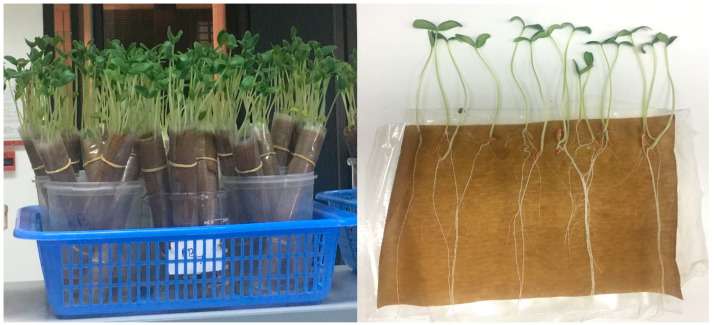
Paper towel seed germination.

Nine (B1, B2, B20, B21, B23, B24, B27, B28, and B29) of the thirty bacterial strains with antagonistic activity and IAA-producing ability were evaluated for growth promotion of cucumber seedlings using the paper towel method in accordance with the International Seed Testing Association [48]. Cucumber seeds were surface sterilized with sodium hypochlorite (1% NaClO, 5 min) and rinsed three times with autoclaved distilled water. The bacterial strains were grown in NB medium with shaking (200 rpm) at 28 °C for 48 h. Then, the bacterial suspension was prepared and adjusted to 10^6^–10^7^ CFU/mL spectrophotometrically at 600 nm wavelength (Thermo Fisher Scientific, Waltham, MA, USA). The sterilized seeds were mixed with a bacterial suspension in a 50 mL flask at room temperature (140 rpm, 3 h). Sterile sieves were used to separate the seeds from the bacterial suspension. After bacterization, 25 seeds were placed in each germination paper and the paper was rolled and placed in a beaker with a suitable amount of sterile distilled water and maintained in a growth chamber at 28 °C for 10 days (Figure 1). Seeds treated only with water served as the control. Eventually, germinated seeds, and root and shoot lengths were measured to calculate the vigor index using the following formula: Vigor index = (mean shoot length + mean root length) × %Germination [49].

### 2.6. Statistical Analysis

All the experiments were conducted in a completely randomized design with three replications in each treatment and repeated three times. All data were subjected to one-way analysis of variance (ANOVA) and means were compared by the least significant difference test (*p* < 0.05) using the R software (version 4.0.3). The data are presented as means ± standard deviation (SD) and displayed in graphical form.

## 3. Results

### 3.1. Isolation of Endophytic and Rhizospheric Bacteria

Thirty bacterial strains were isolated from the *C. colocynthis* plants. Eleven strains came from the rhizosphere (B1–B11), while nineteen were endophytes from the roots (B12–B18), stems (B19–B22), leaves (B23–B26), and fruits (B27–B30). Some strains were found in only one location, while other strains were found in both locations (Wadi Al Alaq and Wadi Bani Khalid) (Table 1).

### 3.2. Molecular Identification

The obtained nucleotide sequences were deposited in GenBank under defined accession numbers (Table 1). The identity indices of the bacterial strains with their relevant strain on the NCBI database are presented in Table 1. There was up to nearly 100% resemblance detected amongst the strains and sequences in the database (except B16, B20, and B29). Identification of the bacterial strains showed that they belonged to different genera and species. These strains belonged to 10 bacterial genera, namely, *Achromobacter*, *Pantoea*, *Pseudomonas*, *Rhizobium*, *Sphingobacterium*, *Bacillus*, *Sinorhizobium*, *Staphylococcus*, *Cupriavidus*, and *Exiguobacterium* (Table 1). *Pseudomonas*, *Achromobacter*, and *Bacillus* were the most abundant genera. The most abundant species were *Achromobacter xylosoxidans* and *Pseudomonas plecoglossicida*. The bacterial species *Achromobacter xylosoxidans*, *Pseudomonas plecoglossicida*, and *Pantoea dispersa* were detected in the rhizosphere as well as the endosphere of *Citrullus colocynthis* (Table 1).

### 3.3. Antagonistic Activity against F. solani and P. aphanidermatum

**Figure 2:**
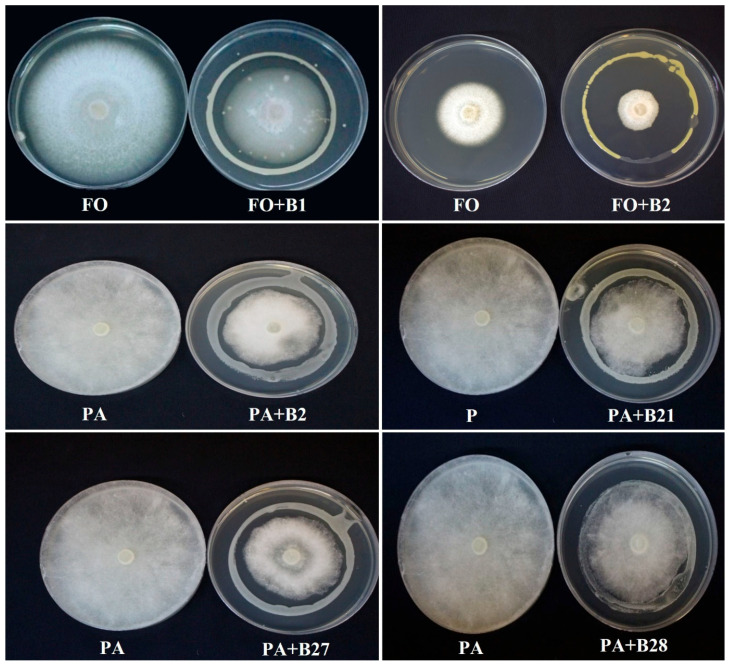
Dual culture assay showing the antifungal activity of selected bacterial strains against *Fusarium solani* (FO) and *Pythium aphanidermatum* (PA). (B) refers to the different bacterial strains.

**Figure 3:**
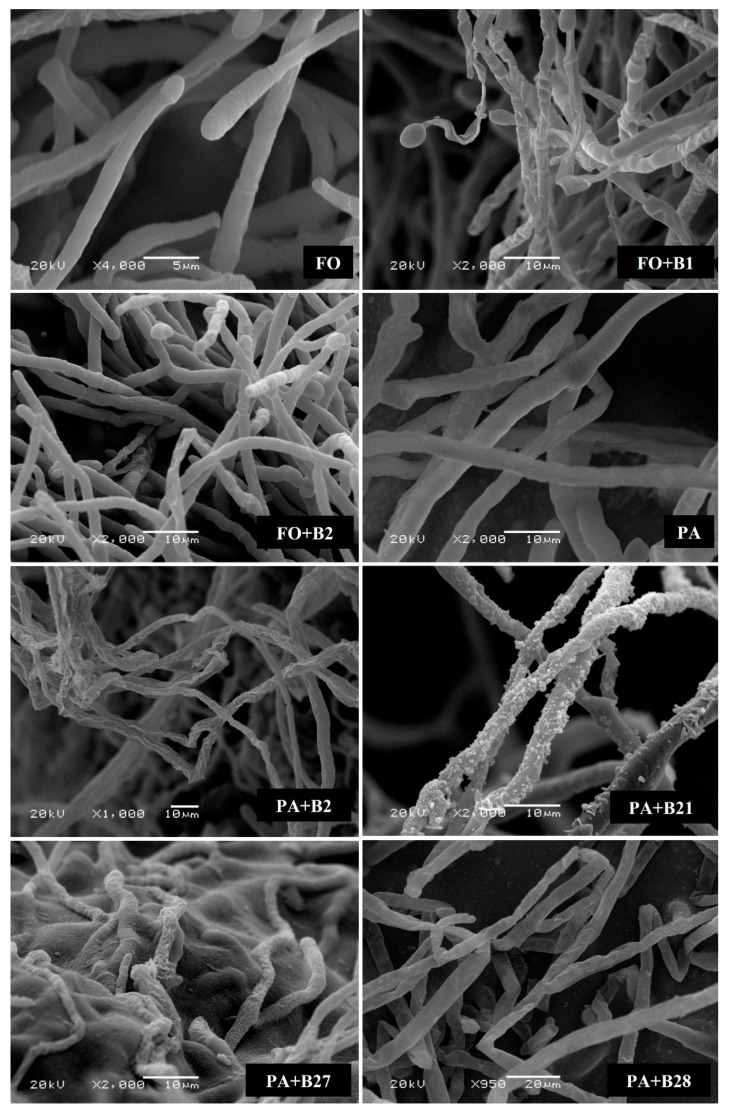
Antagonistic activity of selected bacterial strains on *Fusarium solani* (FO) and *Pythium aphanidermatum* (PA) mycelial morphology depicted using a scanning electron microscope (SEM). Normal patterns of hyphae presented in the control (FS and PA). Distorted mycelial structure, wrinkled or shrunken patterns presented in FO + B1, FO + B2, PA + B2, PA + B21, PA + B27, and PA + B28.

The bacterial strains were examined for their ability to retard the growth of *F. solani and P. aphanidermatum* in vitro using the dual culture technique (Figure 2). Out of the 30 bacterial strains, only 9 rhizospheric and endophytic bacterial strains caused inhibition against *F. solani* growth, while 4 strains inhibited *P. aphanidermatum* growth. Among these strains, only four of the strains demonstrated an inhibitory effect toward the growth of both fungal pathogens. SEM observation of *F. solani* and *P. aphanidermatum* hyphae at the inhibition zone showed negative effects of the selected bacterial strains on the morphology of the pathogens. The bacterial strains caused shrinking and deformation of their hyphae, indicative of a loss in turgidity and cellular content (Figure 3).

**Figure 4:**
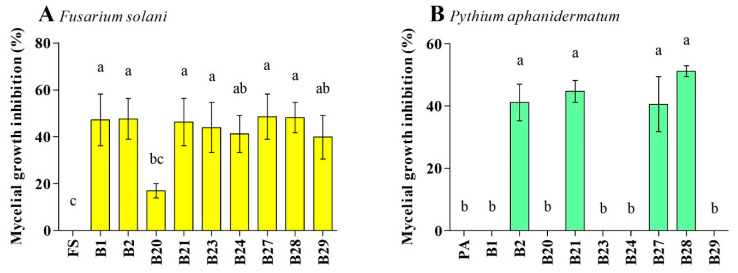
Effect of bacterial antagonists on the inhibition of *Fusarium solani* (FO) (**A**) and *Pythium aphanidermatum* (PA) (**B**) mycelial growths. Values show the means ± standard error (*n* = 3), and significant differences at *p* < 0.05 are indicated by different lowercase letters above the columns.

The nine antagonistic strains belonged to *Achromobacter xylosoxidans*, *Pantoea dispersa*, *Bacillus cereus*, *Pseudomonas stutzeri*, *Staphylococcus gallinarum*, and *Exiguobacterium indicum*. These strains hindered the growth of *F. solani* and *P. aphanidermatum* with different percentages of inhibition. The inhibition percentage of *F. solani* and *P. aphanidermatum* growth by the active strains was in the range of 16.9–48.5% and 40.5–51.2%, respectively. Strains B27 (48.5%) and B28 (48.1%) caused the highest percentage of inhibition toward *F. solani*, while strains B21 (44.7%) and B28 (51.1%) caused the highest percentage of inhibition toward *P. aphanidermatum* (Figure 4). Strains B2, B21, B27, and B28 were inhibitory toward both pathogens, and the inhibition percentage caused by these strains ranged from 41.1 to 51.2%.

### 3.4. Growth-Promoting Assays

#### 3.4.1. Determination of IAA Production

The HPLC method was applied to determine the IAA generated by the selected bacterial strains. Production of IAA (ppm) was detected in all of the selected bacterial strains, comprising B1 (749.1), B2 (660.3), B20 (714.4), B21 (512.9), B23 (699.6), B24 (706.7), B27 (568.7), B28 (581.2), and B29 (732.9). Strain B1 produced the highest amount of IAA, followed by B29. The lowest amount of IAA was produced by the B21 strain.

#### 3.4.2. Effect of Bacterial Strains on Plant Growth Parameters

**Figure 5:**
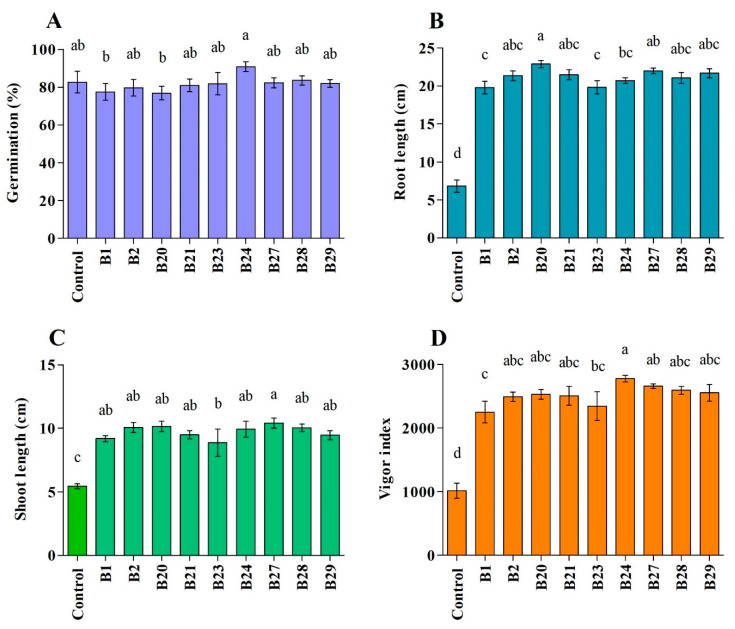
Growth-promoting impact of selected bacterial strains on cucumber plants. Values show the means ± standard error (*n* = 3), and significant differences at *p* < 0.05 are indicated by different lowercase letters above the columns. The subfigures indicate % germination (**A**), root length (**B**), shoot length (**C**), and vigor index (**D**).

All of the tested strains promoted the growth of cucumber, as evidenced by the enhanced seedling vigor compared to the control. The seeds’ germination percentage varied among all strains, ranging from 76.7 to 90.7%. The bacterial strains B24 and B20 were the most active in promoting germination (90.7%) and root growth (22.8%), respectively. In addition, B27 and B24 resulted in the highest shoot length (10.4%) and seedling vigor (63.4%), respectively (Figure 5).

## 4. Discussion

*Pythium aphanidermatum* and *Fusarium solani* are major soilborne pathogens of vegetable crops, especially cucumber, in Oman and different parts of the world, causing up to 100% mortality [5,11,16,50,51,52,53]. Although different methods are used for the control of *Pythium* and *Fusarium*, biological control using antagonistic microorganisms has received more attention during recent years. *Pseudomonas fluorescens* [54,55,56], *P. aeruginosa* [22,28,57], *P. resinovorans* [32], *Lysobacter* sp. [58], *Bacillus cereus* [23,59], *B. subtilis* [25,60,61,62], and *Serratia marcescens* [8,34] have shown antagonistic potential against several soilborne pathogens.

In this study the endophytic bacterial strain *Exiguobacterium indicum* (B28) inhibited *F. solani* and *Pythium aphanidermatum* growth by up to 48.1% and 51.1%, respectively, in a dual culture assay. *Exiguobacterium* species have been used industrially in many applications, such as enzyme production and bioremediation. There are some reports that have indicated the ability of *Exiguobacterium sp.* in promoting plant growth [23,63]. It has been reported that *E. acetylicum* inhibited the growth of *Rhizoctonia solani*, *Sclerotium rolfsii*, *Pythium*, and *Fusarium solani* [64]. In a previous study, Al-Hussini, et al. [65] reported that *E. indicum* strain D1/8 inhibited the growth of *P. aphanidermatum* in vitro and reduced the incidence of damping-off of tomato by 13%. The strain of *E. indicum* (B28) isolated in this study was very effective in inhibiting *F. solani* and *P. aphanidermatum* growth.

The bacterial strain *Achromobacter xylosoxidans* (B1) inhibited *F. solani* by 47.1%. *A. xylosoxidans* has been reported as a biocontrol agent against *F. solani* associated with melon wilt [66]. *A. xylosoxidans* has also been found to increase yields and alleviate drought stress in some crops [67,68]. Another study showed that *A. xylosoxidans* can be used in the control of root-knot nematodes and improve the growth of eggplants [69].

*Pantoea dispersa* (B2) showed high inhibitory activity (~47%) against *F. solani* and *P. aphanidermatum*. It has been reported as a biocontrol agent of sweet potato black rot [70]. *P. dispersa* also proved efficacious in managing Fusarium wilt of pigeon pea [71]. In addition, *P. dispersa* has also been found to stimulate the growth of different crops [72,73,74]. However, no previous reports are available on the use of *P. dispersa* against *F. solani* or *P. aphanidermatum*.

The mycelial growth inhibition of *F. solani* and *P. aphanidermatum* in the dual culture assay plate in this study might be due to diffusible antimicrobial compounds released by the antagonistic bacteria into the agar medium [29]. The differences in the antagonistic potential among the bacterial strains against *F. solani* and *P. aphanidermatum* might be related to the type of antimicrobial compounds produced by them and the sensitivity of the pathogens.

The bacterial strains caused various morphological abnormalities to *F. solani* and *P. aphanidermatum* hyphae, which could be related to the secretion of antifungal metabolites that damage the mycelial cell wall [29,75,76,77]. Hydrolytic enzymes such as cellulase, chitinase, glucanase, and protease are examples of antifungal metabolites that can cause deformations in fungal mycelium [78,79,80,81,82]. *Trichoderma* [78,79,83], *Aspergillus* [29], and *Talaromyces* [13,84] have been reported to produce cellulase enzymes as one of their biocontrol mechanisms. It is evident from this study that the antagonistic bacterial strains induced shrinkage and deformation of the hyphae of the test pathogens. These findings are similar to those reported by Al-Daghari, et al. [36], who recorded shrinkage of the hyphae of *Monosporascus cannonballus* due to the antagonistic effects of *Pseudomonas* spp. The shrinkage of hyphae indicates cell membrane damage and leakage of cytoplasmic contents. Alterations in the intracellular osmotic pressure often result in hyphal distortion.

The bacterial strains improved the growth of cucumber seedlings. The bacterial strain *Achromobacter xylosoxidans* (B1) resulted in the improvement of cucumber growth. Several endophytes and rhizosphere bacteria have been characterized as growth-promoting bacteria since they stimulate root development, improve water and mineral uptake, and produce IAA. These include *Bradyrhizobium*, *Alcaligenes*, *Azoarcus*, *Acetobacter*, *Pseudomonas*, *Enterobacter*, and *Xanthomonas* [85,86,87,88,89,90,91,92,93]. *Achromobacter xylosoxidans* was reported as a growth-promoting bacterium in rice plants [67,94]. The growth-promoting activity of this bacterium could be explained by the high production of IAA, high nitrogenase activity, and P-solubilization. In our study, the high IAA production by this bacterium could be the reason for its high efficacy in promoting cucumber growth. Our study also showed that the antagonistic activity and the growth-promoting activity of the bacteria were different, which may indicate that there is no relationship between the two parameters.

## 5. Conclusions

This study revealed that bacterial strains isolated from *C. colocynthis* have the potential, as biocontrol agents, to prevent damage caused by *F. solani*, *P. aphanidermatum*, and other phytopathogens to vegetable crops, and enhance plant growth. The strains *Pantoea dispersa* and *Exiguobacterium indicum* showed the most antifungal activity against *F. solani*. The mycelial growth of *P. aphanidermatum* was suppressed significantly by *Bacillus cereus* and *Exiguobacterium indicum*. This is the first study to report the efficacy of these bacterial strains from *C. colocynthis* against *F. solani* and *P. aphanidermatum*. Future studies will be required to examine the efficiency of these strains under field conditions, and to prepare their stable bioformulations.

## Figures and Tables

**Table 1:** Identification of rhizospheric and endophytic bacteria associated with *Citrullus colocynthis*.

Bacterial Code	Source of Isolation	Identified Species	Max. Identity (%)	GenBank Accession No.	Place of Collection
B1	Soil	*Achromobacter xylosoxidans*	99.79%	JQ724537	Wadi Al Alaq
B2	Soil	*Pantoea dispersa*	100%	MN725743	Wadi Al Alaq
B3	Soil	*Pseudomonas lini*	99.75%	MT102313	Wadi Al Alaq
B4	Soil	*Pseudomonas plecoglossicida*	99.71%	MF574326	Wadi Al Alaq
B5	Soil	*Pseudomonas aeruginosa*	100%	JQ659890	Wadi Al Alaq
B6	Soil	*Pseudomonas fluorescens*	100%	HM439968	Wadi Al Alaq
B7	Soil	*Rhizobium pusense*	100%	MK734334	Wadi Al Alaq
B8	Soil	*Sphingobacterium spiritivorum*	100%	KR349259	Wadi Bani Khalid
B9	Soil	*Pseudomonas plecoglossicida*	100%	MF574326	Wadi Bani Khalid
B10	Soil	*Achromobacter xylosoxidans*	99.82%	MK537386	Wadi Bani Khalid
B11	Soil	*Pseudomonas putida*	100%	KU672371	Wadi Bani Khalid
B12	Root	*Bacillus aryabhattai*	100%	MT078622	Wadi Al Alaq
B13	Root	*Sinorhizobium meliloti*	99.85%	MT197365	Wadi Al Alaq
B14	Root	*Bacillus anthracis*	98.89%	KF601916	Wadi Al Alaq
B15	Root	*Bacillus anthracis*	99.66%	KF601916	Wadi Bani Khalid
B16	Root	Unknown			Wadi Bani Khalid
B17	Root	*Achromobacter mucicolens*	99.48%	MT534143	Wadi Bani Khalid
B18	Root	*Achromobacter xylosoxidans*	98.79%	MN889379	Wadi Bani Khalid
B19	Stem	*Achromobacter xylosoxidans*	99.86%	MK332530	Wadi Al Alaq
B20	Stem	Unknown			Wadi Al Alaq
B21	Stem	*Bacillus cereus*	100%	MK648340	Wadi Bani Khalid
B22	Stem	*Pseudomonas plecoglossicida*	99.86%	MF574326	Wadi Bani Khalid
B23	Leaf	*Pseudomonas stutzeri*	97.34%	KY606628	Wadi Al Alaq
B24	Leaf	*Staphylococcus gallinarum*	99.93%	MH542297	Wadi Al Alaq
B25	Leaf	*Cupriavidus gilardii*	99.50%	AY860225	Wadi Bani Khalid
B26	Leaf	*Achromobacter xylosoxidans*	100%	MK537386	Wadi Bani Khalid
B27	Fruit	*Pantoea dispersa*	100%	MN725743	Wadi Al Alaq
B28	Fruit	*Exiguobacterium indicum*	99.86%	KT986092	Wadi Al Alaq
B29	Fruit	Unknown			Wadi Bani Khalid
B30	Fruit	*Achromobacter xylosoxidans*	98%	HQ288926	Wadi Bani Khalid

## Data Availability

Sequences were deposited in GenBank under the accession numbers listed in Table 1.
